# Simulation-Based Training for Ultrasound-Guided Central Venous Catheter Placement in Pediatric Patients

**DOI:** 10.15766/mep_2374-8265.11276

**Published:** 2022-09-27

**Authors:** Ryan J. Good, Danielle Mashburn, Erika Jekich, Kristen Miller, Matthew K. Leroue, Jason Woods, Angela S. Czaja

**Affiliations:** 1 Assistant Professor, Department of Pediatrics, Section of Pediatric Critical Care Medicine, University of Colorado Anschutz Medical Campus; 2 Senior Instructor, Department of Pediatrics, Section of Pediatric Critical Care Medicine, University of Colorado Anschutz Medical Campus; 3 Research Instructor, Department of Pediatrics, University of Colorado Anschutz Medical Campus; 4 Assistant Professor, Department of Pediatrics, Section of Emergency Medicine, University of Colorado Anschutz Medical Campus; 5 Associate Professor, Department of Pediatrics, Section of Pediatric Critical Care Medicine, University of Colorado Anschutz Medical Campus

**Keywords:** Central Venous Catheter, Clinical/Procedural Skills Training, Pediatric Critical Care Medicine, Pediatric Emergency Medicine, Pediatrics, Simulation, Ultrasound Skills

## Abstract

**Introduction:**

Central venous catheter (CVC) placement in pediatric patients is lifesaving but associated with complications. To standardize training and decrease complications, we developed a simulation-based ultrasound-guided CVC placement training program for pediatric critical care providers.

**Methods:**

We implemented our CVC placement training program with several groups of learners, including pediatric critical care medicine (PCCM) fellows, pediatric emergency medicine fellows, and PCCM advanced practice providers. Learners completed prework assignments and a knowledge test before participation. The session started with group activities including a learner-led CVC site-selection debate and a team-based competition to list the steps in CVC placement. Next, the learners rotated between four stations for deliberate practice on separate components of CVC placement. Finally, they performed CVC placement on a task trainer. Evaluation included assessment of learner confidence, a knowledge test, and measurement of procedure time before and after training.

**Results:**

Twenty-seven learners participated in the training. Learner confidence in CVC placement increased significantly after participation (median confidence level: 1.5 vs. 4.0, *p* < .001). Learner CVC knowledge also increased significantly after participation (median test score: 68% vs. 88%, *p* < .001). CVC placement procedure time, a marker for skill in CVC placement, decreased significantly after participation (median procedure time: 264 seconds vs. 146 seconds, *p* < .001).

**Discussion:**

Our simulation-based training program effectively increased knowledge, skill, and confidence in CVC placement for a variety of learners. Future work should evaluate the optimal frequency and structure of maintenance training and the impact of training on clinical outcomes.

## Educational Objectives

By the end of this activity, learners will be able to:

1. Explain the indications, anatomy, positioning, and complications of central venous catheter (CVC) placement as demonstrated by participation in the CVC site debate and completion of a knowledge test.
2. List all key steps of CVC placement included in the CVC placement checklist.
3. Demonstrate sterile technique during CVC placement as demonstrated by proper use of a sterile gown, gloves, and ultrasound (US) probe cover.
4. Demonstrate the skills necessary for out-of-plane US-guided CVC placement as demonstrated by cannulating a phantom blood vessel on a task trainer.
5. Perform the steps of the Seldinger technique during CVC placement on a task trainer.

## Introduction

Central venous catheter (CVC) placement in pediatric patients can be lifesaving since CVCs facilitate medication delivery, hemodynamic monitoring, blood sampling, and therapeutic procedures.^1^ As a result, CVC utilization is common in the pediatric intensive care unit (PICU), with a central-line utilization ratio of 0.54 central-line days per PICU patient day.^2^ Although necessary, CVC placement is associated with numerous risks, including damage to surrounding structures, catheter-associated bloodstream infection, and venous thromboembolism (VTE).^3^ Simulation-based training and ultrasound (US) guidance during the procedure decrease the rate of complications during CVC placement.^4,5^

The pedagogical framework for procedural skill training is evolving from “see one, do one, teach one” to a more rigorous process of “learn, see, practice, prove, do, maintain.”^6^ A key element of this framework is summative assessment of a procedural skill on a task trainer prior to performance of the procedure on a patient. Simulation training in CVC placement training is well described and more effective than traditional approaches to CVC placement training.^7–9^ Simulation training in CVC placement also addresses competition among learners for limited procedural opportunities.^10^ Competition for CVC placement experience is heightened in pediatrics, a field with minimal opportunity for training in vascular access during residency training.^11,12^

US guidance during CVC placement offers numerous advantages, including optimization of site selection, visualization of the needle tip during vessel cannulation, and confirmation of the location of the CVC.^13^ US guidance increases success in CVC placement, decreases the rate of complications, and, as a result, is now the standard of care for CVC placement in adults.^14^ US guidance provides similar benefits during CVC placement in pediatric patients but is more challenging due to the smaller size of the central veins in children.^15,16^ Thus, our educational resource focuses on teaching pediatric providers the technique of US guidance during CVC placement using a simulation-based approach.

The intended audience for our resource is any pediatric provider responsible for CVC placement. This includes pediatric residents, fellows, attending physicians, and advanced practice providers (APPs) in pediatric subspecialities such as pediatric critical care medicine (PCCM) and pediatric emergency medicine (PEM). Other simulation-based CVC placement training programs exist, but few address the unique considerations of pediatric CVC placement.^7,17–19^ Specifically, our resource summarizes the current evidence for CVC size selection in pediatric patients, an important factor in preventing catheter-associated VTE. A previous educational resource has outlined the process of internal jugular CVC placement in pediatric patients.^20^ However, little consensus on the optimal site for CVC placement in children currently exists, so our resource features training on the three primary sites for CVC placement: the femoral, internal jugular, and subclavian veins.

## Methods

### Curricular Context

The curriculum was created by the first author, a PCCM attending with expertise in US, with input from other experienced PCCM attendings and PCCM APPs. The curriculum was structured around simulation of CVC placement on a task trainer and designed to include multiple active learning components to encourage participant engagement with the material and achieve the learning objectives. Since CVC placement is a complex task, the skill was broken down into smaller steps to allow learners time for deliberate practice in each of the steps.

We implemented our educational resource within numerous curricular contexts. Initially, we developed the simulation-based training for PCCM fellows during prefellowship orientation, later expanding it to other learners, including PEM fellows and PCCM APPs. Participants had a variety of backgrounds and experience with CVC placement. Most PCCM and PEM fellows had little to no experience in CVC placement, while some of these learners and most of the PCCM APPs had previously performed CVC placement on pediatric patients. To ensure a baseline of knowledge about CVC placement prior to participation in the simulation-based training session, we asked participants to complete the following prework, assigned 1 week before the session:

• Review the CVC study guide (Appendix A).
• Review the schedule for the simulation-based training session (Appendix B).
• Prepare for the CVC site-selection debate (Appendix C).
• Compete the CVC knowledge test (Appendices D and E).
• Watch selected videos on CVC placement (optional).^21,22^

Experts in CVC placement, including attending physicians in PCCM and PEM, senior PCCM fellows, and senior PCCM APPs, facilitated the sessions. To standardize the instruction provided to learners, facilitators reviewed the CVC study guide (Appendix A) prior to the simulation-based training session.

### Implementation

We scheduled the simulation-based training session for 3 hours for novice learners. For sessions with learners who had completed previous simulation-based training or had previous clinical experience in CVC placement, we shortened the session schedule to 2 hours. We maintained a facilitator-to-learner ratio of 1:2 for each session.

#### Introduction

To begin the simulation-based training session, the session leader outlined the schedule (Appendix B), reviewed the learning objectives, and answered questions about the prework (Appendices D and E).

#### CVC site-selection debate

For this activity, each learner presented their argument for their assigned anatomic site for CVC placement. We provided learners with instructions for the activity via email (Appendix C) and encouraged them to use the CVC study guide (Appendix A) to aid in preparation. We preferentially assigned the internal jugular and femoral sites as they are the most common ones for CVC placement at our institution. For sessions with more than two or three learners, the session leader combined learners into groups to work collaboratively on their argument for site selection. To start the debate, the session leader selected one of the anatomic sites and asked the learner(s) to give their 5-minute presentation on the anatomic site utilizing the whiteboard for illustration as necessary. Topics required to be covered by the presenter included (1) anatomy, (2) patient position, (3) technique, and (4) benefits specific to the anatomic site. If the learner(s) failed to cover a topic, the session leader asked questions about the topic that were answered by the presenter or other learners. At the conclusion of the presentation, other learners had an opportunity to ask questions about the anatomic site that were answered by the presenter and/or session leader. Each anatomic site was covered in the same way as the first. At the conclusion of the debate, the session leader answered any remaining questions about the anatomic sites of CVC placement. In our experience, these debates ran well, with little prompting needed from the facilitator. The major pitfall we encountered was an incomplete presentation by the learner, which we addressed by asking specific questions about the topics. If the learner was unable to answer the questions, we allowed other learners in the session to contribute and pointed out where to find the answers in the CVC study guide (Appendix A).

#### Steps of CVC placement activity

The session leader instructed the learners to write down each step of CVC placement in order. After learners completed their lists independently, they paired with another learner to compare their lists and collaboratively develop a shared list of steps in CVC placement. Each group of learners wrote its final list of steps on a piece of paper or a dry-erase board. The instructor then presented the actual list of CVC steps, as outlined in the steps of CVC placement document (Appendix F), with a printed handout or projector. This document was created by PCCM attendings at our institution based on our local practices. A validated task-specific checklist for CVC placement in children had been published but did not include sufficient detail for initial training of novices.^23^ To make this activity competitive, the facilitator assigned points to each team for steps on its shared list that also appeared on the actual list of CVC steps. The facilitator used this exercise to clarify specific steps of CVC placement with the learners before proceeding to the hands-on portion of the session. We removed this activity from the schedule for learners with prior experience in CVC placement.

#### Deliberate practice for components of CVC placement

For this activity, we divided the steps of CVC placement into different components and assigned a facilitator for each component at four hands-on stations. We instructed facilitators to review the CVC study guide (Appendix A) as preparation for facilitating their assigned station. We assigned one facilitator to each station, but up to two learners rotated together through each station depending on the number of learners in the session. At station 1, the learners reviewed the anatomy and positioning for each site of CVC placement. The facilitator used either a mannequin or whiteboard to demonstrate the anatomy and positioning. At station 2, learners reviewed setup and sterile procedure for CVC placement. Learners referenced a list of CVC supplies (Appendix G) and found the location of the supplies in a procedure cart. Facilitators reviewed sterile technique, including skin preparation, use of the sterile US probe cover, gowning, and gloving. Learners then practiced maintaining sterile technique while putting on a sterile gown, gloves, and US probe cover. At station 3, leaners practiced the steps of the Seldinger technique on a CVC placement task trainer. We utilized the same CVC placement equipment that learners would use in the clinical setting. After practicing the Seldinger technique, the learners practiced securing the CVC to the skin using a needle drive, suture, and a banana. At station 4, learners utilized vascular access task trainers and a US machine to practice US-guided vascular access. Facilitators focused the practice at this station on dynamic needle tracking in the out-of-plane technique. Learners could start at any station and rotate through all four stations. We removed the deliberate practice stations from the schedule for learners with prior experience in CVC placement.

#### CVC placement on task trainer

In the final portion of the simulation-based training session, learners practiced CVC placement from start to finish on a task trainer mannequin. We utilized a commercially available CVC task trainer, but other low-cost options for CVC placement training also exist.^16^ Facilitators provided feedback and coaching during the CVC placement attempts and ensured the learners completed each step in CVC placement. If equipment, specifically the task trainer and US machines, was not sufficient to allow each learner to practice CVC placement at the same time, we instructed the other learners to continue deliberate practice with US guidance for vascular access. With this additional time, facilitators reviewed the in-plane approach to US-guided vascular access.

#### CVC placement clinical vignettes

To provide additional training in CVC placement after completion of the simulation-based training session, we developed clinical vignettes for CVC placement (Appendix H). Facilitators used the vignettes with learners to describe a clinical scenario of a pediatric patient in need of CVC placement. Learners responded to the vignette by answering questions about optimal CVC site and size selection. Next, they demonstrated CVC placement technique on the task trainer mannequin. Facilitators worked one-on-one with learners during these sessions, which lasted approximately 30 minutes.

### Evaluation Strategy

To evaluate the effectiveness of the CVC placement simulation-based training session, learners completed a 17-question multiple-choice knowledge test before and after the session (Appendices D and E). The knowledge test was developed using the content covered in the CVC study guide (Appendix A). Learners also reported their level of confidence with CVC placement before and after the session using a Likert scale (1 = *not confident at all,* 5 = *extremely confident*). To evaluate learner skill in CVC placement, we measured the time required to perform CVC placement on the task trainer before and after the session. Evaluation data were collected on a convenience sample of learners who participated in the training sessions.

## Results

A total of 27 learners participated in eight separate simulation-based CVC placement training sessions. Learners included eight PCCM fellows, eight PEM fellows, and 11 PCCM APPs. Participation in previous simulation-based training in CVC placement was reported by 52% of the learners, while 59% reported any previous CVC placement in a patient. Among CVC placement sites, the femoral site was the most reported (59%), followed by the internal jugular (37%) and subclavian (4%). Among learners who reported previous CVC placement experience in patients, most (81%) reported fewer than five experiences. The first author, a PCCM attending, led each session with assistance from additional facilitators, including two senior PCCM APPs, four senior PCCM fellows, and two other PCCM attendings.

**Figure 1.**
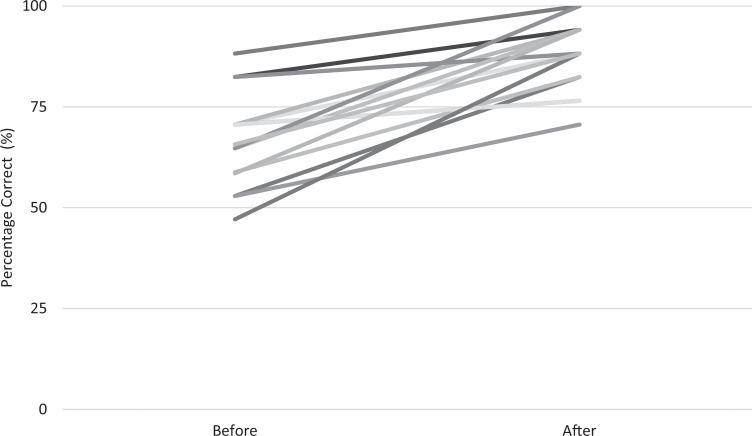
Percentage of questions answered correctly on the knowledge test before and after participation in the simulation-based training session (*p* < .001 by Wilcoxon matched-pairs signed rank test, *n* = 16).

**Figure 2.**
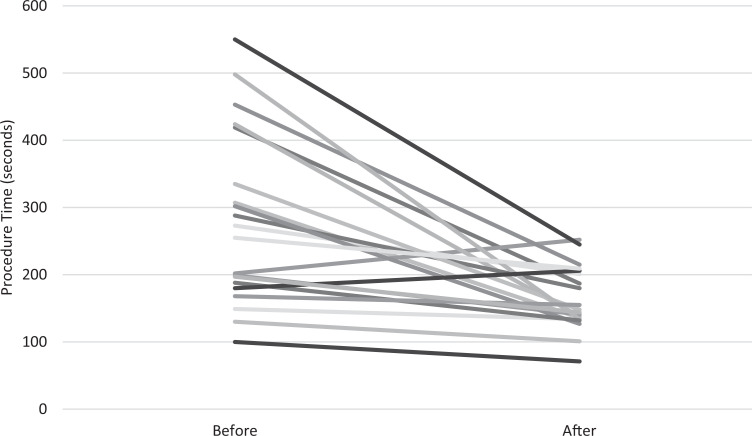
Central venous catheter procedure time, in seconds, before and after participation in the simulation-based training session (*p* < .001 by Wilcoxon matched-pairs signed rank test, *n* = 20).

Learner performance on the CVC placement knowledge test increased significantly after participation, from a median score of 68% before (interquartile range [IQR]: 59%-80%) to a median score of 88% after (*p* < .001 by Wilcoxon matched-pairs signed rank test, *n* = 16; Figure 1). Learner confidence with CVC placement also increased significantly after participation, from a median confidence level of 1.5 before (IQR: 1.0-2.0) to a median confidence level of 4.0 after (IQR: 3.0-4.0, *p* < .001 by Wilcoxon matched-pairs signed rank test, *n* = 16). Learner skill in CVC placement also improved significantly after participation, from a median procedure time of 264 seconds before (IQR: 182-398 seconds) to a median procedure time of 146 seconds after (IQR: 134-205 seconds, *p* < .001 by Wilcoxon matched-pairs signed rank test, *n* = 20; Figure 2). Assessment data were collected from a convenience sample of learners, as the knowledge tests and confidence assessments were voluntary. We did not perform measurement of CVC placement procedure time on all learners.

## Discussion

This simulation-based, US-guided CVC placement training program for pediatric critical care providers improved learner knowledge, skill, and comfort with a low-frequency but potentially lifesaving procedure in PCCM. We incorporated established educational strategies in CVC placement training, including active learning, deliberate practice, and simulation, when we developed our curriculum. However, the resources we created have a unique focus on pediatric patients, with special attention to catheter size selection and consideration of the risks and benefits of each anatomic site for CVC placement. Originally designed for PCCM fellows, our training program has been implemented with a wide variety of learners, including PCCM APPs and PEM fellows, with similar positive results, thus demonstrating the portability of the training program across settings and with learners of different backgrounds and experience with CVC placement.

We modeled our curriculum on previously published CVC placement training programs for emergency medicine, anesthesia, and internal medicine residents that utilized simulation-based training and deliberate practice for individual steps of CVC placement.^17,19^ However, the training in these previous examples was distributed over three to four separate sessions, whereas our curriculum was conducted during a single 2- to 3-hour session. Learners in our curriculum gained skill in CVC placement despite the lack of distributed sessions. This concentrated approach to training has the added benefit of being more feasible for learner and facilitator schedules. While most CVC placement training programs focus on novice learners, over half of the learners participating in our curriculum had previously placed a CVC in a patient. Despite this clinical experience, learners who were not novices still benefited from simulation-based training with improvement in knowledge, comfort, and skill. Other simulation-based training programs have demonstrated similar benefits for experienced learners.^7,16^ Frequent opportunities for simulation-based training are vital for low-frequency procedures, as a time lag of more than 2 weeks between simulation and performance of a procedure on a patient has been identified as a barrier to CVC placement skill transfer from simulation to patient encounters.^24^ This finding further supports the need for ongoing maintenance training, especially within pediatrics, a specialty with fewer procedural opportunities.^11,12^

This educational resource reflects the evolution of our CVC placement training program over several years, with the introduction of new facilitators, new activities, and learners of different backgrounds. First designed for novice learners, the curriculum has been modified for learners who are not novices by shortening the session length and removing the deliberate practice stations. We also found that some learner groups, specifically PEM fellows, were more comfortable with the US guidance skill required for CVC placement. Thus, we shortened the portion of the session focusing on US guidance and emphasized other portions including anatomic site selection and catheter size selection. The CVC clinical vignettes were designed for PCCM fellows and APPs with significant CVC placement selection. In the future, we plan to incorporate CVC placement on the simulation task trainer into multidisciplinary simulation scenarios to reflect the true clinical environment in which CVC placement occurs and increase the cognitive load for learners experienced in CVC placement.

We acknowledge that our educational resource is limited as it was conducted at a single center and with a relatively small number of learners. Additionally, our resource depends on skilled facilitators and specialized equipment to implement the simulation-based training sessions. Our evaluation approach also has several limitations. We did not utilize a validated CVC placement checklist for evaluation of CVC placement skill, instead assessing skill by measuring procedure time. Several validated CVC placement checklists, including one designed specifically for pediatric critical care, have been published that could be used by those who implement our training program.^23,25^ We also did not perform follow-up evaluation of learners after the training session to assess for retention and the impact of clinical experiences with CVC placement. This information could be beneficial for determining the need for additional maintenance simulation-based training. Finally, we did not systematically collect qualitative feedback from learners or facilitators, which would have better informed iterative cycles of improvement in the sessions. Future work in CVC placement training should focus on determining the optimal frequency and structure of maintenance training as well as evaluating the impact of training on the clinical outcomes of CVC placement.

## Appendices


CVC Study Guide.docxCVC Session Schedule.docxCVC Email Instructions.docxCVC Knowledge Test.docxCVC Knowledge Test Answer Key.docxSteps of CVC Placement.docxCVC Equipment.docxCVC Clinical Vignettes.docx
*All appendices are peer reviewed as integral parts of the Original Publication.*
