# Supplementary material for: Simulation-Based Training for Ultrasound-Guided Central Venous Catheter Placement in Pediatric Patients
Source: MedEdPORTAL. 2022 Sep 27;18:11276. doi: 10.15766/mep_2374-8265.11276 (PMC9512948; doi:10.15766/mep_2374-8265.11276)
Supplement: Supplementary file 1 — CVC Study Guide.docxCVC Session Schedule.docxCVC Email Instructions.docxCVC Knowledge Test.docxCVC Knowledge Test Answer Key.docxSteps of CVC Placement.docxCVC Equipment.docxCVC Clinical Vignettes.docx [file mep_2374-8265.11276-s001.zip › B. CVC Session Schedule.docx]

**Central Venous Catheter (CVC) Simulation-Based Training Session**

**Goal:** Upon completion of this workshop, learners will have the knowledge and skills necessary to perform CVC placement.

**Learning Objectives:**

1. Explain the indications, anatomy, positioning, and complications of central venous catheter (CVC) placement as demonstrated by participation in the CVC site debate and completion of a knowledge test.
2. List all key steps of CVC placement included in the CVC placement checklist.
3. Demonstrate sterile technique during CVC placement as demonstrated by proper use of a sterile gown, gloves, and ultrasound (US) probe cover.
4. Demonstrate the skills necessary for out-of-plane US guided CVC placement as demonstrated by cannulating a phantom blood vessel on a task trainer.
5. Perform the steps of Seldinger technique during CVC placement on a task trainer.

**Schedule:**

- Introduction (5 minutes)
- Group Activity #1: CVC Site Selection Debate (20 minutes)
  - Sites: femoral, internal jugular, subclavian
  - Equipment: whiteboard, dry erase marker
- Group Activity #2: Steps of CVC placement (20 minutes)
  - Equipment: paper, pens, “Steps of CVC Placement”
- Hands-on Activities (60 minutes)
  - 1 learner per session and 15 minutes per session

| **Station** | **Equipment** | **Activities** |
| --- | --- | --- |
| 1. Anatomy and positioning | - White board - Markers - Task trainer or mannequin | - Draw relevant anatomy on white board - Demonstrate positioning with task trainer or mannequin |
| Set-up and sterile procedure | - Procedure cart - CVC equipment checklist - Sterile gown - Sterile gloves - Sterile US probe cover | - Identify equipment needed - Review skin preparation - Demonstrate sterile gown and gloving |
| Seldinger technique | - CVC placement kit - Task trainer - Suture - Needle driver - Scissors - Banana | - Review equipment in CVC placement kit - Practice steps of Seldinger technique on task trainer - Practice suturing catheter to the “skin” with a banana |
| US guidance | - US machine - Task trainer - Needle - Syringe | - Practice US guidance for cannulation of a phantom blood vessel on a task trainer - Focus on out-of-plane (short axis) approach |

- Complete CVC Placement (60 minutes)
  - Each learners completes 1 central line placement from start to finish on task trainer
  - Other learners practice US guided vessel cannulation on task trainers including the in-plane (“long axis”) approach
