# Supplementary material for: Simulation-Based Training for Ultrasound-Guided Central Venous Catheter Placement in Pediatric Patients
Source: MedEdPORTAL. 2022 Sep 27;18:11276. doi: 10.15766/mep_2374-8265.11276 (PMC9512948; doi:10.15766/mep_2374-8265.11276)
Supplement: Supplementary file 1 — CVC Study Guide.docxCVC Session Schedule.docxCVC Email Instructions.docxCVC Knowledge Test.docxCVC Knowledge Test Answer Key.docxSteps of CVC Placement.docxCVC Equipment.docxCVC Clinical Vignettes.docx [file mep_2374-8265.11276-s001.zip › C. CVC Email Instructions.docx]

**E-Mail Instructions for Central Venous Catheter (CVC) Site Debate**

**The following e-mail text was sent to learners prior to participation in the simulation-based CVC training session:**

*As part of the CVC training session, we will hold a friendly debate about which anatomic site of CVC placement is the “best”. Each learner will be assigned to an anatomic site as listed below. Please prepare a 5-minute presentation that covers the anatomy, position and technique for your assigned anatomic location and attempt to make the case for why your location is the best. Use of drawings or illustrations is highly encouraged!*

*Femoral: [insert learner name(s) here]*

*Internal Jugular: [insert learner name(s) here]*

*Subclavian: [insert learner name(s) here]*
