# Supplementary material for: Simulation-Based Training for Ultrasound-Guided Central Venous Catheter Placement in Pediatric Patients
Source: MedEdPORTAL. 2022 Sep 27;18:11276. doi: 10.15766/mep_2374-8265.11276 (PMC9512948; doi:10.15766/mep_2374-8265.11276)
Supplement: Supplementary file 1 — CVC Study Guide.docxCVC Session Schedule.docxCVC Email Instructions.docxCVC Knowledge Test.docxCVC Knowledge Test Answer Key.docxSteps of CVC Placement.docxCVC Equipment.docxCVC Clinical Vignettes.docx [file mep_2374-8265.11276-s001.zip › D. CVC Knowledge Test.docx]

**Central Venous Catheter (CVC) Knowledge Test**

Name: __________________________

Date: ___________________________

1. Have you participated in **ANY** simulation-based training in CVC placement? Yes / No
2. How many CVCs have you placed at the following anatomic sites?

Femoral: ________

Internal Jugular: ________

Subclavian: ________

1. How would you rate your confidence in performing CVC placement?

1 – Not confident at all

2 – A little confident

3 – Somewhat confident

4 – Moderately confident

5 – Extremely confident

1. Which of the following patients is most likely to benefit from placement of a CVC?
   1. A 12-year-old female with severe diabetic ketoacidosis who needs frequent lab draws
   2. A 5-year-old male with bacterial pneumonia and septic shock requiring vasoactive infusions
   3. A 6-month-old female with bronchiolitis who is intubated for respiratory failure and needs continuous pain/sedation infusions
   4. A 14-month-old male with a severe traumatic brain injury who requires a bolus of 3% hypertonic saline for presumed intracranial hypertension
2. Among adult patients who require CVC placement, which anatomic site is associated with the lowest rate of intravascular complications including catheter-related blood stream infection (CLABSI) and venous thromboembolism (VTE)?
   1. Femoral
   2. Internal jugular
   3. Subclavian
   4. None of the above
3. Which anatomic site of CVC placement has the highest rate of pneumothorax?
   1. Femoral
   2. Internal jugular
   3. Subclavian
   4. None of the above
4. Please select the mandatory infection prevention measures employed during CVC placement. (select all that apply)
   1. Full length sterile drape covering the patient
   2. Sterile drape covering at least 50% of the patient
   3. Cap and face mask for all providers in the room
   4. Cap and face mask for the procedure operator only
   5. Full sterile gown and gloves for the procedure operator
   6. Full sterile gown and gloves for all providers in the room
5. What is the minimum time that skin cleaning solution must be applied to the insertion site during femoral CVC placement?
   1. 30 seconds
   2. 60 seconds
   3. 120 seconds
   4. 180 seconds
   5. None of the above
6. Which best describes optimal patient head position during CVC placement in internal jugular vein?
   1. Head of bed at 30$^{\circ}$, head rotated 15$^{\circ}$ towards the ipsilateral shoulder
   2. Head of bed at 30$^{\circ}$, head neutral
   3. Head of bed at 30$^{\circ}$, head rotated 15$^{\circ}$ towards contralateral shoulder
   4. Head of bed at 0$^{\circ}$, head rotated 15$^{\circ}$ towards the ipsilateral shoulder
   5. Head of bed at 0$^{\circ}$, head neutral
   6. Head of bed at 0$^{\circ}$, head rotated 15$^{\circ}$ towards contralateral shoulder
7. When measuring a patient to select the appropriate CVC length for the internal jugular or subclavian vein, which skin landmark should serve as a surrogate for the superior vena cava – right atrium junction?
   1. Mid-point of the right clavicle
   2. Suprasternal notch
   3. Junction of the manubrium and 2^nd^ rib
   4. Junction of the manubrium and 4^th^ rib
   5. None of the above
8. What size CVC should be used for a 4-month-old, 7 kg infant during CVC placement in the internal jugular vein?
   1. 4 French
   2. 5 French
   3. 7 French
   4. None of the above
9. Which bony landmarks should be identified prior to CVC placement in the femoral vein?
   1. Anterior iliac spine
   2. Greater trochanter of the femur
   3. Pubic symphysis
   4. B and C
   5. A and C
   6. All of the above
10. Which answer has the correct relationship of the vein to the artery at each anatomic site?
    1. Femoral – lateral; internal jugular – lateral
    2. Femoral – lateral; internal jugular – medial
    3. Femoral – medial; internal jugular – lateral
    4. Femoral – medial; internal jugular – medial
11. Which ultrasound probe should be used for ultrasound guidance during CVC placement?
    1. Phased array
    2. Curvilinear
    3. Linear
    4. None of the above
12. Which answer correctly pairs the direction of needle advancement at each anatomic site?
    1. Femoral – umbilicus; subclavian – suprasternal notch
    2. Femoral – pubic symphysis; subclavian – suprasternal notch
    3. Femoral – umbilicus; subclavian – junction of manubrium and 2^nd^ rib
    4. Femoral – pubic symphysis; subclavian – junction of manubrium and 2^nd^ rib
13. During an initial attempt to place a femoral CVC with ultrasound guidance, the provider does not cannulate the vein despite advancing the needle 3 cm. On ultrasound, the needle tip appears medial to the femoral vein. What should the provider do next?
    1. Remove the needle and attempt CVC placement at another site
    2. Retract the needle until the needle tip is just above the femoral vein, then move the needle laterally and re-advance needle
    3. Retract the needle until the needle tip is just below the skin surface, then move the needle laterally and re-advance the needle
    4. Leave the needle in place and attempt to cannulate the vein with an angiocatheter
    5. None of the above
14.
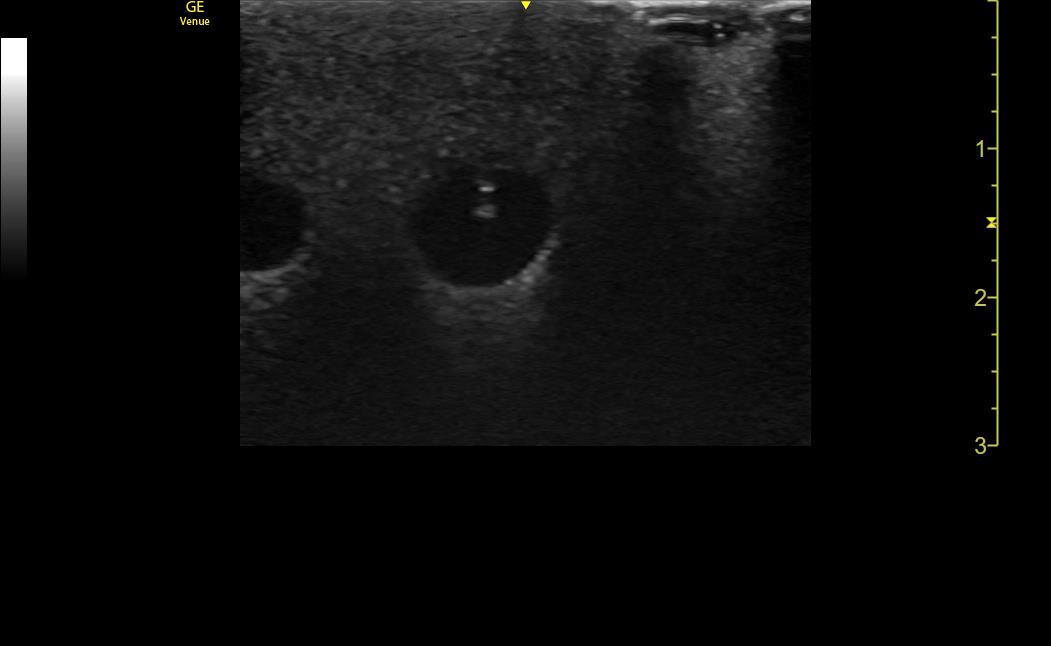
During an initial attempt at femoral CVC placement, the provider captures the above transverse image of the femoral vein. Approximately how deep is the needle tip in this image?
    1. 1.5 cm
    2. 2.6 cm
    3. 3.4 cm
    4. Not enough information to determine depth
15. During an attempt to place a CVC in the right internal jugular vein, the provider aspirates bright red, pulsatile blood from the introducer needle. What should the provider do next?
    1. Insert the guidewire and continue with CVC placement
    2. Remove the needle and hold pressure for 10-15 minutes
    3. Leave the needle in place and call vascular surgery
    4. Leave the needle in place and obtain a chest x-ray to confirm the needle location
    5. None of the above
16. What is the “gold standard” for confirmation of CVC position prior to use?
    1. X-ray
    2. Ultrasound
    3. Qualitative assessment
    4. Pressure transduction
    5. None of the above
17. When can ultrasound be utilized during CVC placement?
    1. Evaluate vein patency prior to site selection
    2. Real-time guidance during needle advancement
    3. Visualization of the guidewire prior to passing the dilator
    4. Visualization of CVC after placement
    5. All of the above

***All images and figures within this document were created by the authors***
