# Supplementary material for: Simulation-Based Training for Ultrasound-Guided Central Venous Catheter Placement in Pediatric Patients
Source: MedEdPORTAL. 2022 Sep 27;18:11276. doi: 10.15766/mep_2374-8265.11276 (PMC9512948; doi:10.15766/mep_2374-8265.11276)
Supplement: Supplementary file 1 — CVC Study Guide.docxCVC Session Schedule.docxCVC Email Instructions.docxCVC Knowledge Test.docxCVC Knowledge Test Answer Key.docxSteps of CVC Placement.docxCVC Equipment.docxCVC Clinical Vignettes.docx [file mep_2374-8265.11276-s001.zip › E. CVC Knowledge Test Answer Key.docx]

**Central Venous Catheter (CVC) Knowledge Test – Answer Key**

1. B

2. C

3. C

4. A, C, E

5. C

6. F

7. C

8. A

9. E

10. C

11. C

12. A

13. C

14. D

15. B

16. A

17. E
