# Supplementary material for: Simulation-Based Training for Ultrasound-Guided Central Venous Catheter Placement in Pediatric Patients
Source: MedEdPORTAL. 2022 Sep 27;18:11276. doi: 10.15766/mep_2374-8265.11276 (PMC9512948; doi:10.15766/mep_2374-8265.11276)
Supplement: Supplementary file 1 — CVC Study Guide.docxCVC Session Schedule.docxCVC Email Instructions.docxCVC Knowledge Test.docxCVC Knowledge Test Answer Key.docxSteps of CVC Placement.docxCVC Equipment.docxCVC Clinical Vignettes.docx [file mep_2374-8265.11276-s001.zip › F. Steps of CVC Placement.docx]

**Central Venous Catheter (CVC) Placement Steps**

| **Critical Steps** | **Yes** | **No** | **Comments** |
| --- | --- | --- | --- |
| Patient/parent educated about the need for procedure and risks, including bleeding, clots, and infection |  |  |  |
| Equipment assembled (see list) |  |  |  |
| Sedation and monitoring chosen, and person dedicated to sedation |  |  |  |
| Procedural time-out |  |  |  |
| Patient positioned for line chosen |  |  |  |
| Patient measured for appropriate line length and size |  |  |  |
| US to ensure patency of vessel |  |  |  |
| Skin prep with chlorhexidine |  |  |  |
| Skin prep allowed to dry |  |  |  |
| Patient covered with drape |  |  |  |
| Catheter flushed and all lumens clamped |  |  |  |
| US used to identify vessel |  |  |  |
| Introducer needle inserted in skin then vessel  (Syringe attached to needle, bevel up, numbers on syringe lined up with bevel) |  |  |  |
| Hand firmly holding introducer needle and syringe removed |  |  |  |
| Advance guide wire (Need full control over wire at ALL times, may place in circular configuration to help) |  |  |  |
| Remove needle |  |  |  |
| US to confirm location of guidewire in vessel |  |  |  |
| Nick the skin, sliding blade along wire, with blade upwards off wire |  |  |  |
| Thread dilator along wire |  |  |  |
| Thread catheter over the wire, wire emerges from distal lumen |  |  |  |
| Remove wire |  |  |  |
| Draw and flush each lumen, then clamp them |  |  |  |
| Place caps on each lumen |  |  |  |
| Suture catheter to skin |  |  |  |
| Thoroughly clean skin |  |  |  |
| Apply sterile dressing |  |  |  |
| X-ray ordered to verify placement |  |  |  |
| Once position confirmed, notify nurse that line may be used |  |  |  |
| Properly document line placement in chart |  |  |  |
