# Supplementary material for: Simulation-Based Training for Ultrasound-Guided Central Venous Catheter Placement in Pediatric Patients
Source: MedEdPORTAL. 2022 Sep 27;18:11276. doi: 10.15766/mep_2374-8265.11276 (PMC9512948; doi:10.15766/mep_2374-8265.11276)
Supplement: Supplementary file 1 — CVC Study Guide.docxCVC Session Schedule.docxCVC Email Instructions.docxCVC Knowledge Test.docxCVC Knowledge Test Answer Key.docxSteps of CVC Placement.docxCVC Equipment.docxCVC Clinical Vignettes.docx [file mep_2374-8265.11276-s001.zip › G. CVC Equipment.docx]

**Central Venous Catheter (CVC) Equipment**

| **Supplies** | **Yes** | **No** |
| --- | --- | --- |
| Sterile gown |  |  |
| Sterile towels |  |  |
| Sterile gloves |  |  |
| Facemask |  |  |
| Surgical cap |  |  |
| Skin cleaning solution (e.g., Chloraprep) |  |  |
| Plastic drape |  |  |
| Ultrasound machine |  |  |
| Sterile ultrasound probe cover |  |  |
| Central line kit (appropriate size and number of lumens) |  |  |
| Needle driver |  |  |
| Scalpel |  |  |
| Suture scissors |  |  |
| Suture |  |  |
| NS/heparin for flushes |  |  |
| 4 X 4 gauze |  |  |
| Appropriate size sterile dressing |  |  |
| 25g needle for lidocaine (optional) |  |  |
