# Supplementary material for: Simulation-Based Training for Ultrasound-Guided Central Venous Catheter Placement in Pediatric Patients
Source: MedEdPORTAL. 2022 Sep 27;18:11276. doi: 10.15766/mep_2374-8265.11276 (PMC9512948; doi:10.15766/mep_2374-8265.11276)
Supplement: Supplementary file 1 — CVC Study Guide.docxCVC Session Schedule.docxCVC Email Instructions.docxCVC Knowledge Test.docxCVC Knowledge Test Answer Key.docxSteps of CVC Placement.docxCVC Equipment.docxCVC Clinical Vignettes.docx [file mep_2374-8265.11276-s001.zip › H. CVC Clinical Vignettes.docx]

**Central Venous Catheter (CVC) Clinical Vignettes**

1. An 8 yo, 25kg, male arrives to the PICU from the ED after presenting following high speed MVC. CT head reveals large left frontotemporal SAH and IPH with associated 2mm rightward midline shift. Chest CT revealed left clavicular fracture and bilateral pulmonary contusions. Abdominal imaging unremarkable. Exam on arrival is notable for GCS 6T after a hypertonic saline bolus. It is deemed that the patient needs a central venous catheter for ongoing hyperosmolar therapy.
   1. What placement site should be avoided? Why? **– avoid the internal jugular (IJ) vein because of the potential risk of impairing cerebral venous drainage with the catheter**

The decision is made to place a subclavian CVC.

- 1. Based on the patients age, what Fr size should be chosen? **– 5 Fr**
  2. How do you determine appropriate catheter length? Where should the tip of the catheter end? **– measure from the insertion site to the clavicular notch and then down to the insertion of the 2^nd^ rib to the manubrium; the catheter tip should be located at the SVC-RA junction**
  3. Describe how to position patient for a subclavian CVC. **– patient supine, head of be at 0° if not contraindicated (i.e., TBI), head neutral, arm adducted, towel roll between scapula parallel to spine to bring shoulder to neutral or posterior position**
  4. What the relationship of the subclavian vein to the subclavian artery? **– vein is anterior to artery**
  5. If multiple PVCs are seen upon guide wire advancement what should be done immediately? **– pull back guide wire until PVCs resolve**

1. A 17 yo, 55kg, female presents to PICU after reported amlodipine overdose. At the outside hospital she was started on high dose norepinephrine via an IO. Toxicology recommended transfer to PICU given possibility that patient would require cannulation onto VA ECMO. On arrival to PICU is on norepinephrine infusion at 0.5 mcg/kg/min and epi infusion at 0.4 mcg/kg/min.
   1. What considerations should be made when choosing placement site in this patient? **– given potential need for VA ECMO cannulation should consider cannulation strategy (cervical vs. femoral) prior to CVC placement**
   2. If a femoral CVC is decided upon what size should be chosen based on patients age? **– 7 Fr**
   3. Describe how to position patient? **– supine, hip flexed and externally rotated, towel roll under hips perpendicular to spine**
   4. What anatomic landmarks should be identified? What is the relationship of the femoral vein to the femoral artery? **– anterior iliac spine, pubic symphysis, inguinal ligament, umbilicus; femoral vein is medial to the femoral artery**
   5. What direction should your needle be directed? **– towards the umbilicus**
   6. Where should the tip of the catheter end? **– between L2 and L5**
   7. What is the gold standard for line confirmation? **– abdominal x-ray for femoral CVC**
2. A 4 yo, 20kg, male with high-risk neuroblastoma undergoing induction chemotherapy is transferred to PICU following RRT with septic shock. Upon arrival to PICU he requires intubation and initiation of vasopressors for persistent hypotension despite adequate volume resuscitation. The decision is made to place an IJ CVC.
   1. What are the specific risks and benefits of placing an IJ CVC in this patient? **– benefits = stable central venous access for medication administration (specifically vasoactive medications), ability to monitor CVP and SvO2; risks = bleeding, infection, arterial puncture, pneumothorax**
   2. What anatomic landmarks should be identified? What is the relationship of the carotid artery and the internal jugular vein? **– clavicle, sternocleidomastoid, nipple; internal jugular vein is lateral to carotid artery**
   3. What are the ways in which you can confirm venous position during attempted venipuncture? **– US visualization, qualitative assessment of blood color and flow, pressure transduction, blood gas analysis**
   4. If you inadvertently puncture the artery with the needle, what should you do next? **– hold pressure for at least 5 minutes**
   5. How far should you insert the dilator? **– the dilator must transverse the soft tissue and reach the vessel; can use US to measure this distance; avoid inserting the dilator all the way to the hub**
