# Supplementary material for: Simulation-Based Training for Ultrasound-Guided Central Venous Catheter Placement in Pediatric Patients
Source: MedEdPORTAL. 2022 Sep 27;18:11276. doi: 10.15766/mep_2374-8265.11276 (PMC9512948; doi:10.15766/mep_2374-8265.11276)
Supplement: Supplementary file 1 — CVC Study Guide.docxCVC Session Schedule.docxCVC Email Instructions.docxCVC Knowledge Test.docxCVC Knowledge Test Answer Key.docxSteps of CVC Placement.docxCVC Equipment.docxCVC Clinical Vignettes.docx [file mep_2374-8265.11276-s001.zip › A. CVC Study Guide.docx]

**Central Venous Catheter (CVC) Study Guide**

1. **Consent**
   1. Risks
      1. General
         1. Bleeding
         2. Hematoma
         3. Arterial puncture (6-8%)
         4. Nerve injury
         5. Malposition
         6. Inability to place (~10%)
         7. Infection
         8. Venous thromboembolism (VTE)
      2. Site specific
         1. **Femoral**
            1. Bowel/bladder puncture
            2. Retroperitoneal hematoma
         2. **Internal Jugular (IJ)** and **Subclavian**
            1. Hemothorax
            2. Pneumothorax
            3. Arrhythmia
            4. Thoracic duct injury
   2. Benefits
      1. Stable intravenous access
         1. Safely administer medications that are vesicants (vasoactive infusions) or hyperosmolar (dextrose concentration > 12.5%, hypertonic saline)
         2. Decrease need for peripheral intravenous (PIV) catheter placement and risk of PIV infiltration or extravasation (PIVIE)
      2. Hemodynamic monitoring
         1. Central venous pressure (CVP)
         2. Mixed venous saturation (S_v_O_2_)
      3. Ability to perform frequent blood sampling
         1. Consider arterial line as alternative
      4. Administer therapies that require large blood flow rates (i.e. dialysis, plasmapheresis)
         1. Requires larger diameter catheter (see section on catheter size selection)
   3. Alternatives
      1. PIV – risk of PIVIE, unable to safely administer all medications
      2. Intra-osseous (IO) line – risk of growth plate injury, compartment syndrome, temporary (< 24 hours), faster to place
      3. Peripherally inserted central catheter (PICC) – smaller diameter and longer line not as useful for rapid fluid administration/resuscitation, not available 24/7, higher rates of VTE and CLABSI
   4. Documentation – document the consent process in the CVC note template; signed consent is NOT required at our institution
   5. Implied consent – acceptable in emergency situations if the patient or parent is not able to provide consent but should be documented in the CVC note template
   6. Follow your unit specific policies for obtaining pre-procedural consent and for procedure documentation
2. **Anatomy**
   1. **ALWAYS** review patient anatomy prior to visualizing vascular structures with ultrasound (US)
   2. **Femoral**
      1. Landmarks - anterior iliac spine, pubic symphysis, inguinal ligament, umbilicus
      2. Vascular relationship - common femoral vein = medial to common femoral artery
         1. “**NAVL**” acronym – **N**erve, **A**rtery, **V**ein, **L**ymphatics
      3. Notes – Femoral artery overlaps the femoral vein in a significant number of patients and overlap may increase with distance from the inguinal ligament


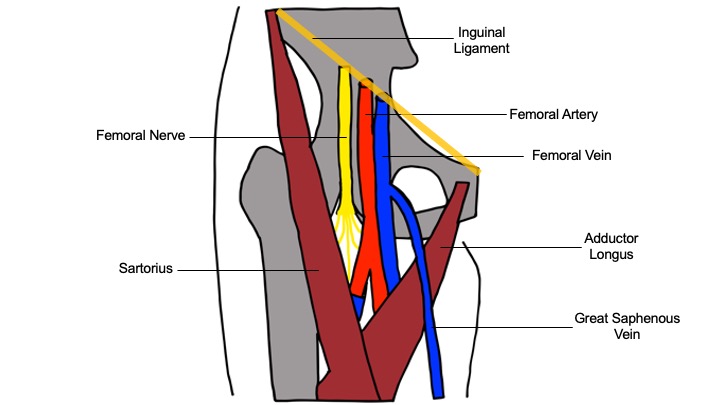


**Figure 1**: Relevant anatomy for femoral vein cannulation

- 1. **IJ**
     1. Landmarks – clavicle, sternocleidomastoid (sternal and clavicular heads), nipple
     2. Vascular relationship – IJ = lateral to carotid artery
     3. Notes –IJ may overlap the carotid artery


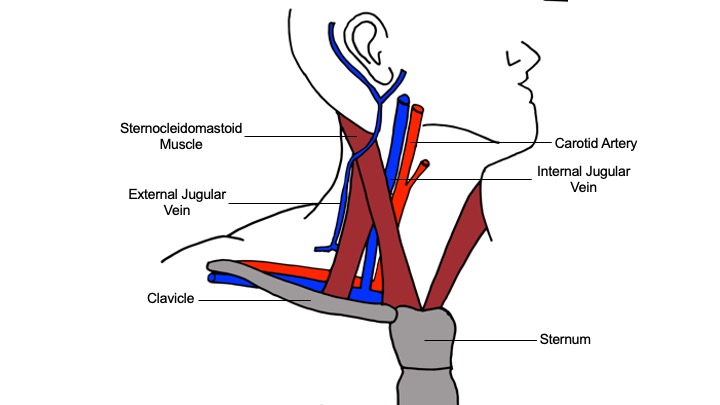


**Figure 2**: Relevant anatomy for internal jugular vein cannulation

- 1. **Subclavian**
     1. Landmarks – medial third of the clavicle, sternum, suprasternal notch
     2. Vascular relationship – subclavian vein = anterior to subclavian artery


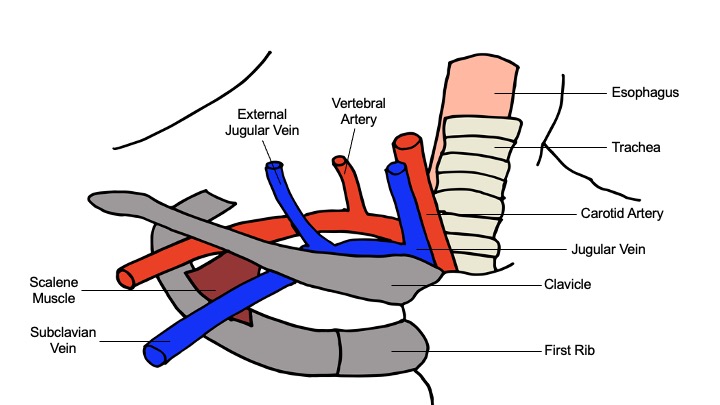


**Figure 3**: Relevant anatomy for subclavian vein cannulation

1. **Preparation**
   1. Patient position
      1.
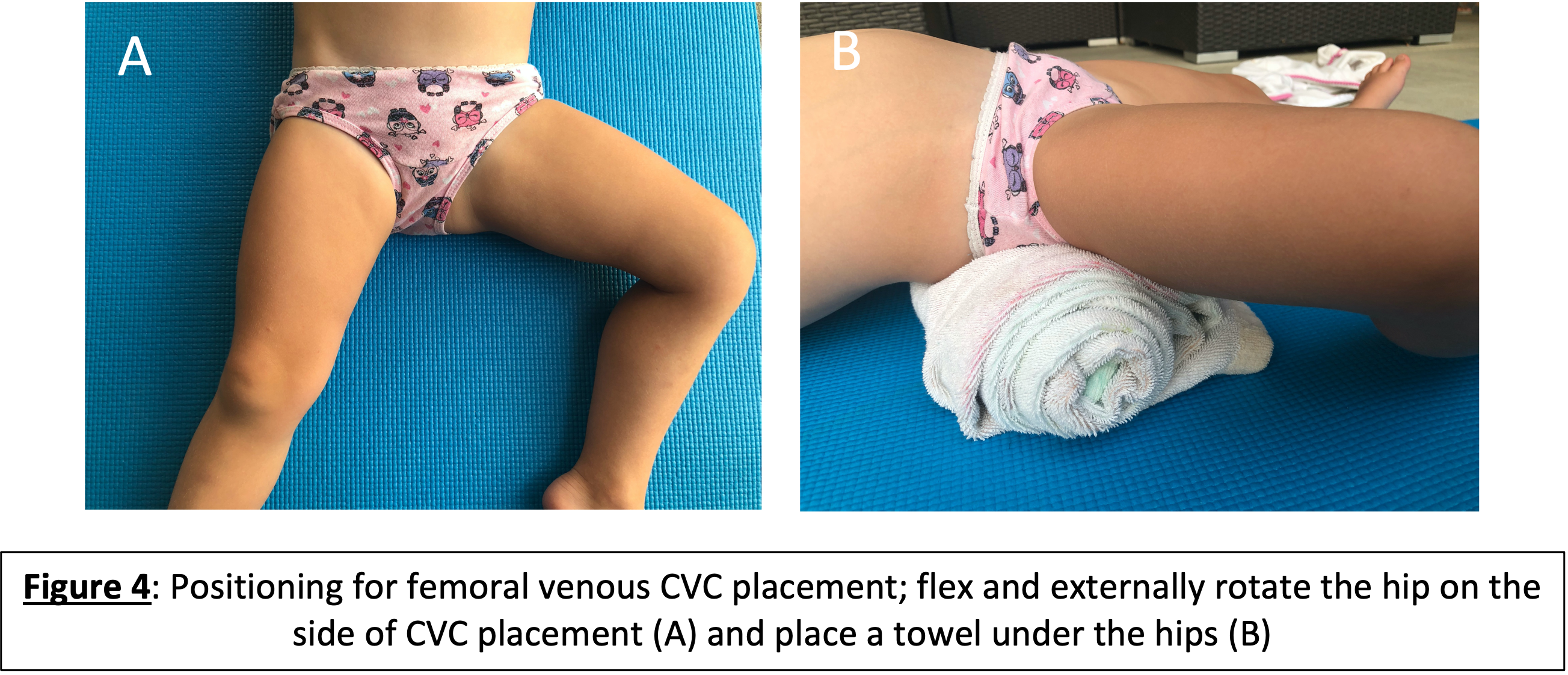
**Femoral** – supine, hip flexed and externally rotated, towel roll under hips perpendicular to spine
      2. **IJ –** supine, HOB at 0$^{\circ}$ (or 15$^{\circ}$ of Trendelenburg position) if not contraindicated (i.e. TBI), head rotated 10-15$^{\circ}$ towards contralateral shoulder – further rotation can decrease the diameter of the IJ and may increase overlap of the internal carotid artery, towel roll under shoulders perpendicular to spine to improve access to the neck but neck extension may decrease the diameter of the IJ


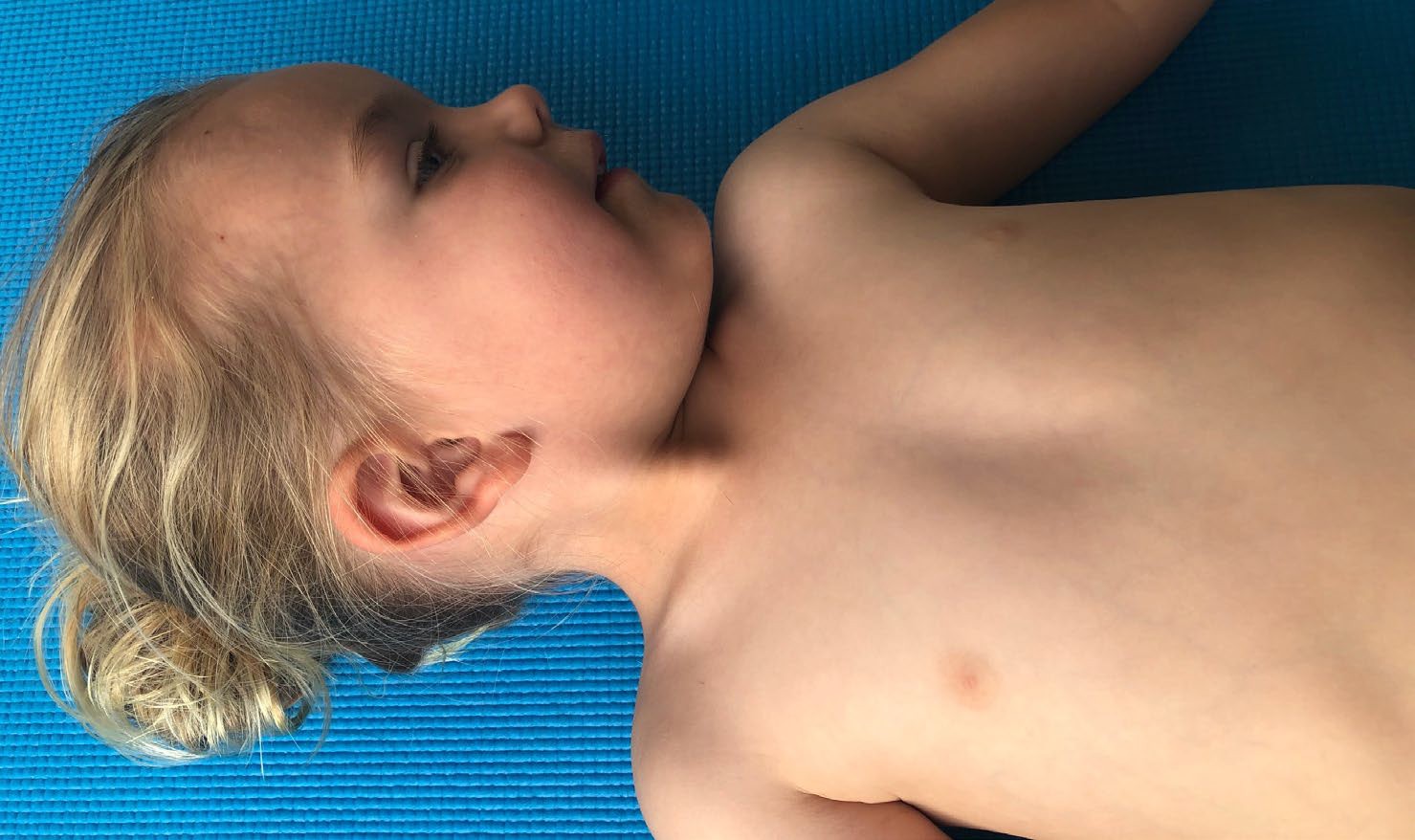


**Figure 5**: Positioning for internal jugular CVC placement with patient supine, head of bed at 0$^{\circ}$, head rotated 10-15$^{\circ}$ to the contralateral shoulder

- - 1. **Subclavian –** supine, HOB at 0$^{\circ}$ if not contraindicated (i.e. TBI), head neutral, arm adducted, towel roll between scapula parallel to spine to bring shoulder to neutral or posterior position

| Age | Catheter Size (Fr) |
| --- | --- |
| < 6 mo | 4 |
| 6 mo – 12 yr | 5 |
| > 12 yr | 7 |

- 1. Equipment – see CVC equipment checklist
  2. Line selection
     1. Diameter (i.e. French)
        1. Outer diameter (mm) = size (Fr)/3
        2. Age-based criteria
           1. Most frequently used but not standardized^1,2^
           2.
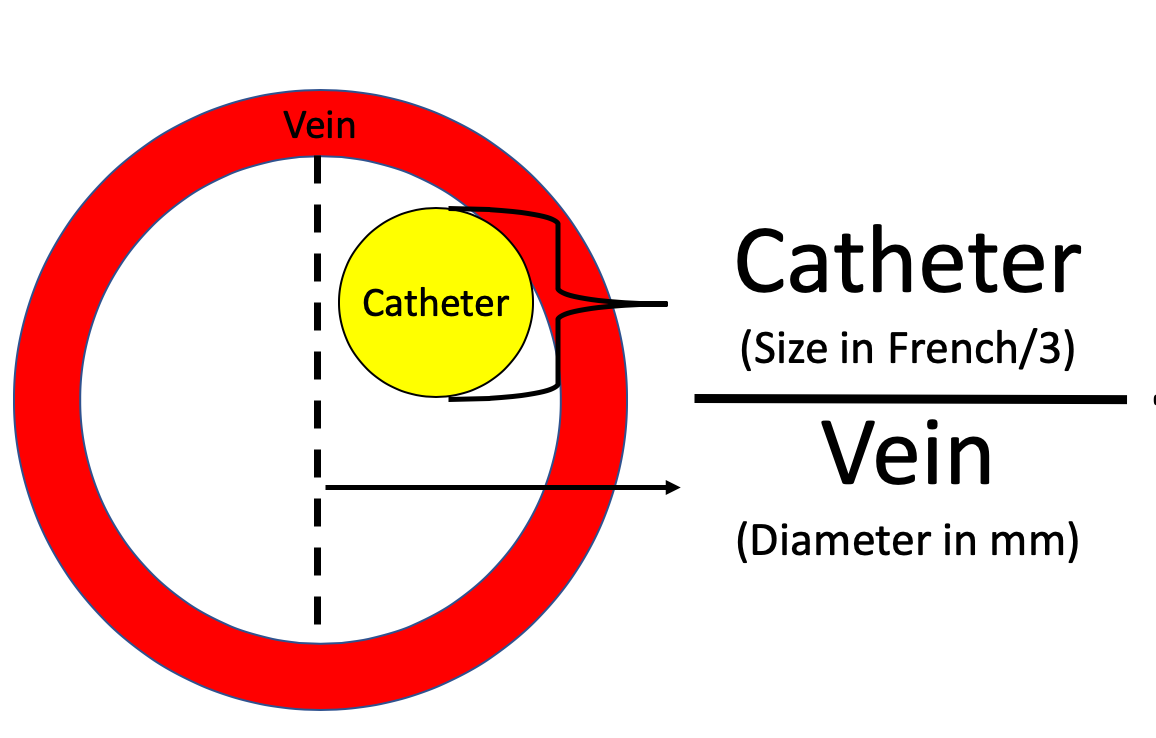
3 Fr single lumen catheter is also available but rarely utilized
        3. Catheter-to-vein (CV) ratio
           1. Higher CV ratio 🡪 decreased blood flow 🡪 potential for increased VTE risk^3^
           2. CV ratio < 0.5 proposed as threshold^4^


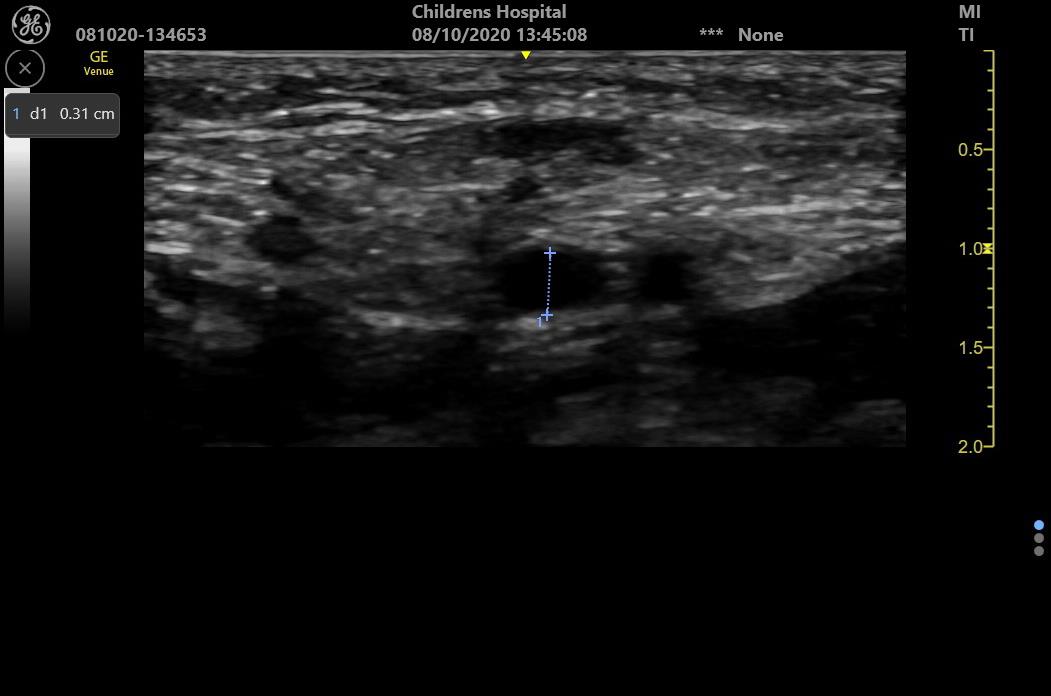


**Figure 6**: Vein diameter measurement in the anterior-posterior plane from inner vessel wall to inner vessel wall

- - 1. Length
       1. **Femoral** – goal = tip in the IVC above the iliac vein confluence but below renal veins (L2)
          1. Measure from insertion point to umbilicus (L3)
          2. Adult patients = 20 cm
          3. Estimation = 8 + (0.45 x weight (kg)) ^5^
       2. **Internal Jugular** – goal = tip just above the SVC-RA junction^6,7^
          1. Chest x-ray (CXR) – measure from insertion site to level of carina
          2. Patient – measure from insertion site to junction of manubrium and 2^nd^ rib
          3. Adult patients = 15 cm (right), 17 cm (left)
          4. Estimation = 8% of height
       3. **Subclavian –** goal = tip just above the SVC-RA junction^8^
          1. CXR – measure from insertion site to clavicular notch + vertical distance from clavicular notch to carina
          2. Patient – measure from insertion site to clavicular notch + vertical distance from notch to junction of manubrium and 2^nd^ rib
          3. Adults patient = 15 cm (right), 18 cm (left)
          4. Estimation = 8% of height (add 1-2 cm for left)


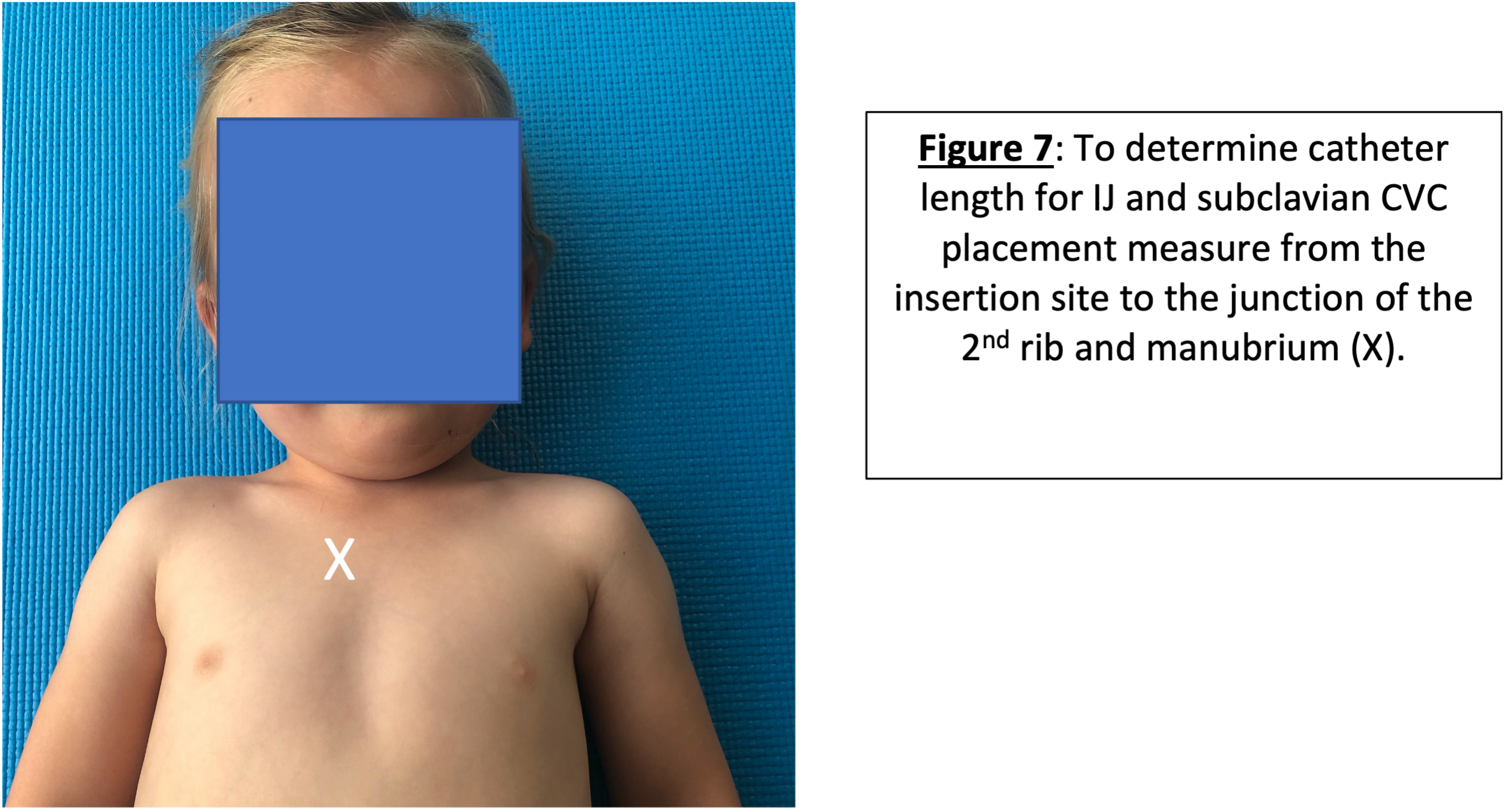


- - 1. Number of lumens – minimum number required; more lumens associated with higher risk of VTE
    2. Dialysis and/or plasmapheresis require specific catheter sizes
       1. Discuss with consulting service (i.e. Nephrology, Transfusion Medicine) to confirm catheter size required
  1. Flush all lumens and caps with saline to minimize the risk of air embolism
  2. Ultrasound (US) set-up
     1. Position US directly across from the proceduralist
     2. Select high-frequency linear probe
     3. Probe held perpendicular to vein location
     4. Use light pressure to avoid compression of the vein
     5. Visualize the potential sites for CVC placement prior to patient preparation to help aid in optimal site selection
     6. Vein identification
        1. Orientation based on normal anatomy
        2. Veins = thin-walled, compressible, non-pulsatile
        3. Confirmation with color Doppler flow


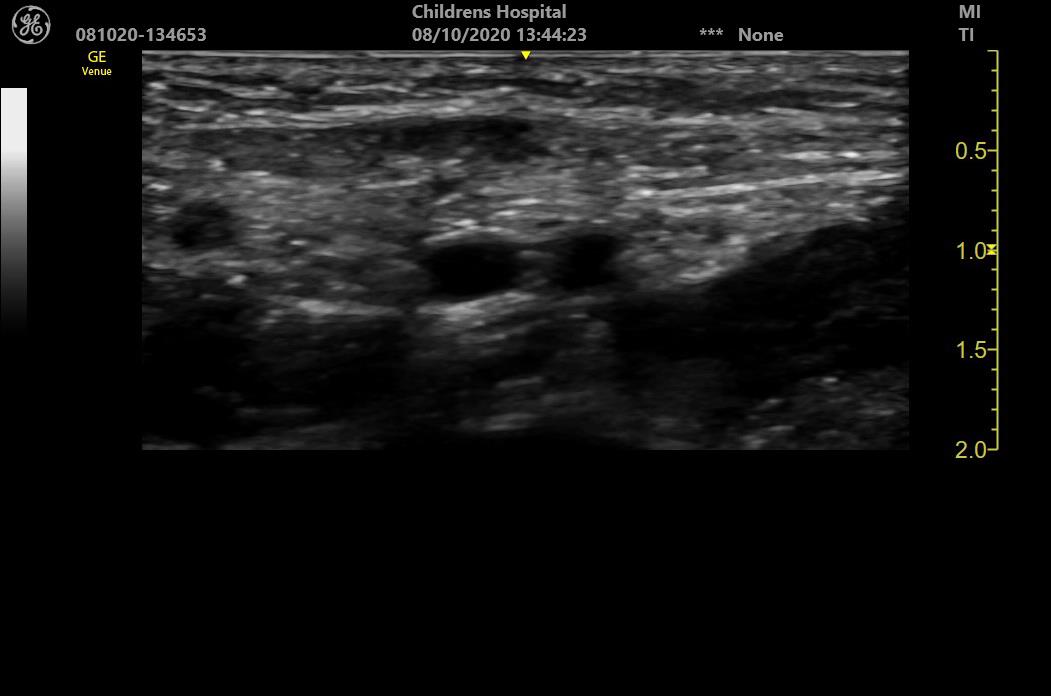

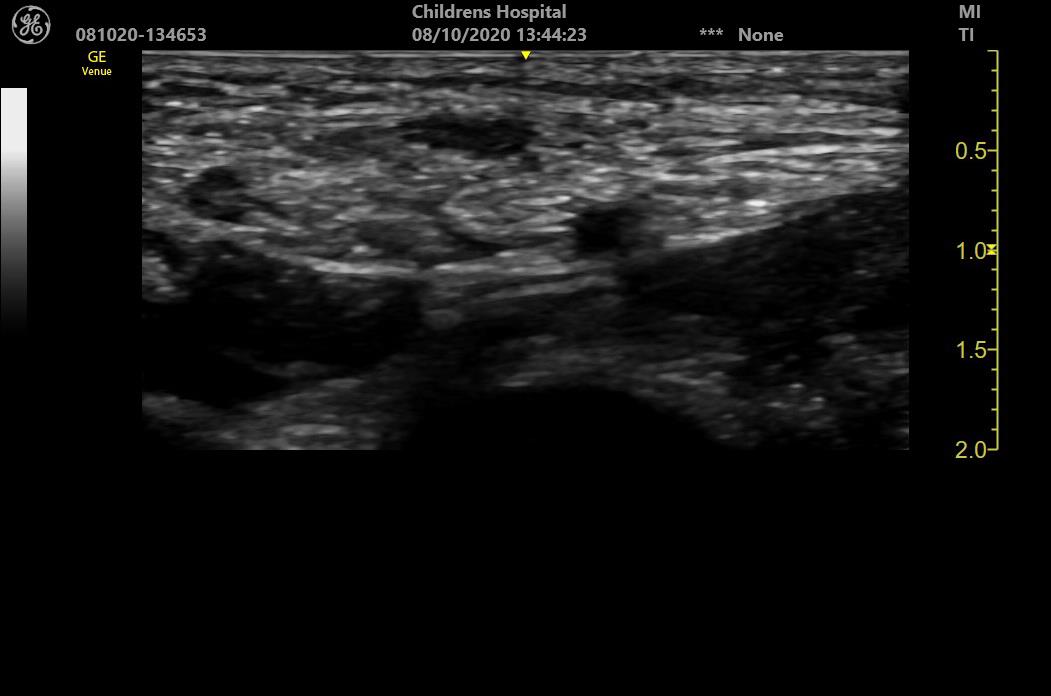


**Figure 8**: Femoral vein (v) and artery (a) in short axis without pressure (A) and with pressure (B) applied by the probe

a

v

v

a

A

B


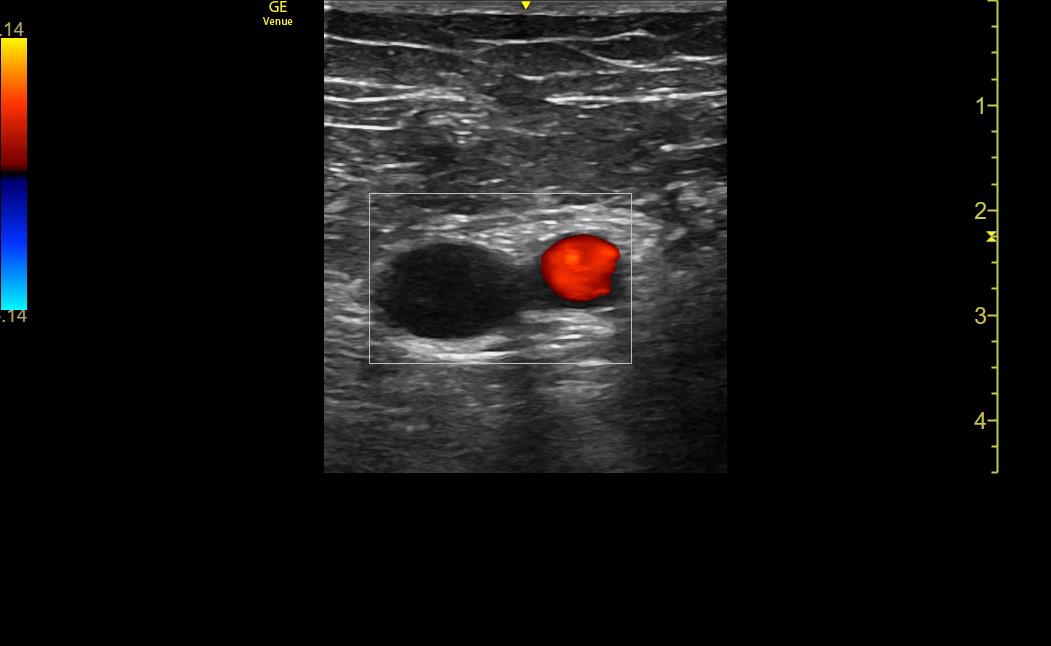


**Figure 9**: Femoral vein and artery in short axis view. Color flow imaging reveals high velocity, pulsatile flow in the artery (white ‘a’) and low/no velocity flow in the vein (white ‘v’)

a

v

- - 1. Follow vein proximally and distally from planned insertion site to determine course and relation to other structures
    2. Adjust depth so that vein is in the middle of display
    3. US image acquisition examples:


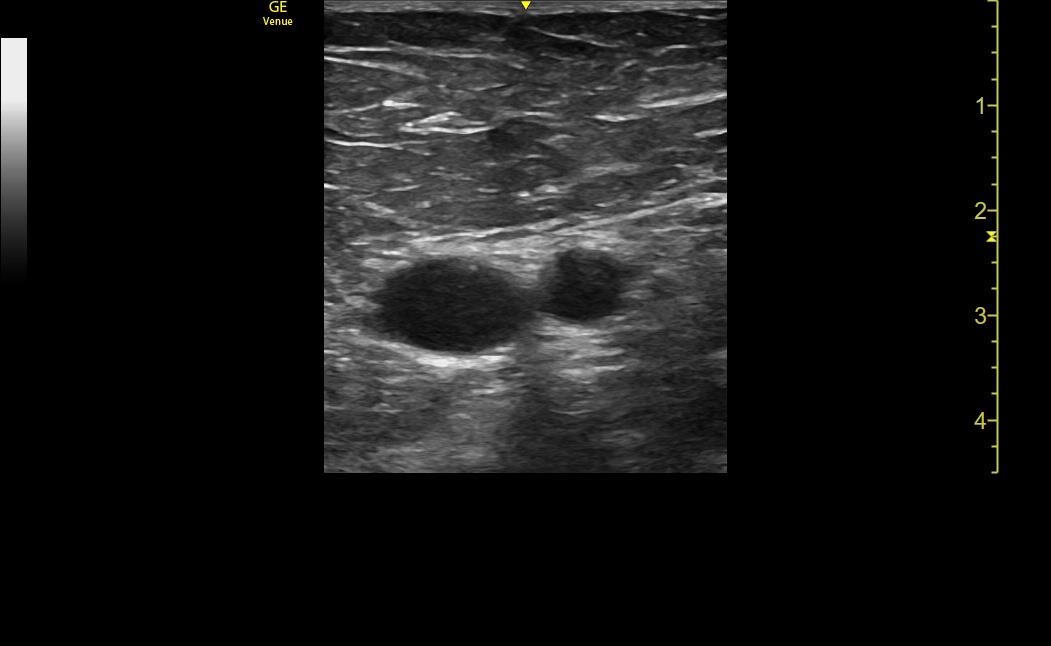


**Figure 10**: Femoral vein and artery in short axis view. Artery (white ‘a’) is lateral to vein (white ‘v’)

a

v

a

v


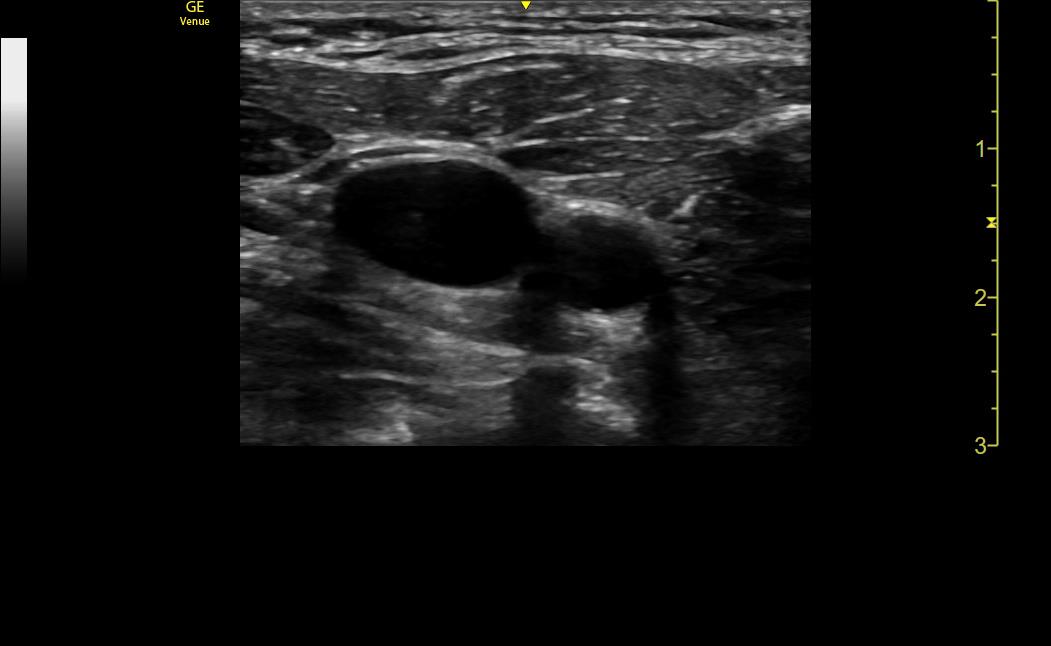


**Figure 11**: Internal jugular vein and internal carotid artery in short axis view. Artery (white ‘a’) is medial to vein (white ‘v’). Sternocleidomastoid muscles (SCM) are superficial.

a

v

SCM

SCM


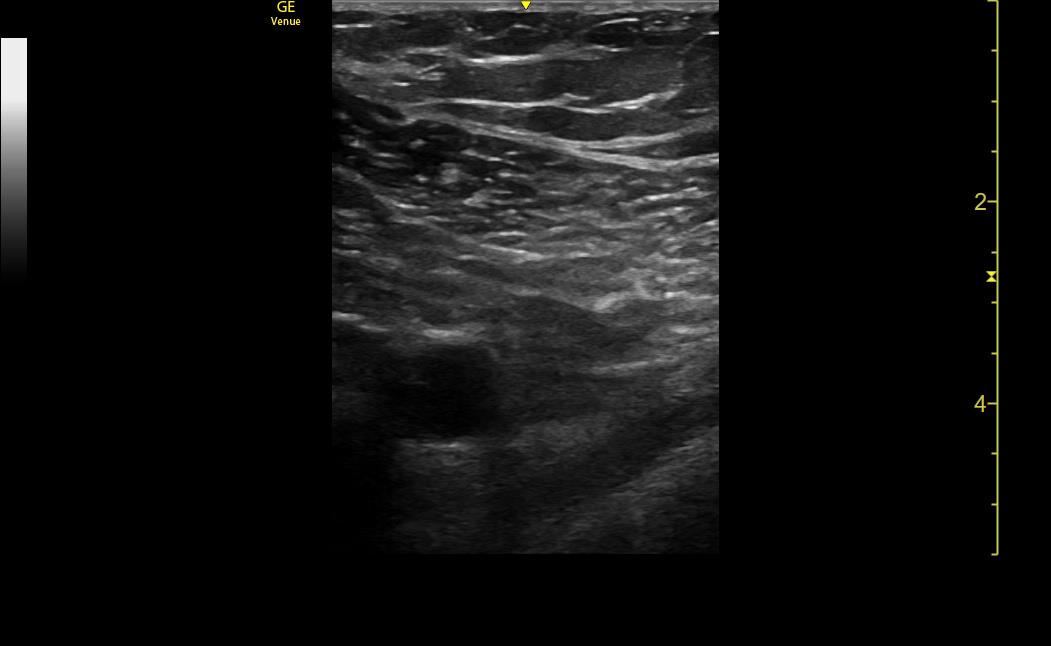


**Figure 12**: Subclavian vein (white ‘v’) in short axis view. Pleura (white ‘p’) is posterior and inferior.

v

p

- 1. Sterile procedure
     1. Proceduralist – gown, gloves, hat, mask
     2. Assistants – hat, mask
     3. Full body draping if possible but ensure access to important sites (i.e. endotracheal tube, PIVs)
     4. Skin preparation = Chlora-prep for 2 minutes and allow to fully dry before skin puncture
     5. US probe cover

1. **Procedure**
   1. US guidance
      1.
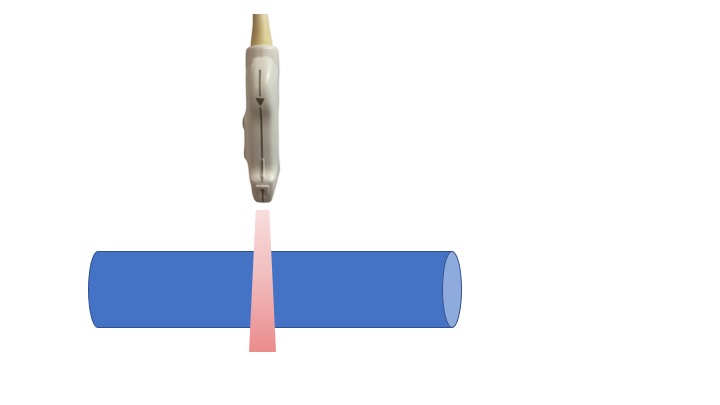
Short axis (out-of-plane, transverse) approach
         1. Probe “leads” needle towards the vein
            1. Pivot or slide probe **AWAY** from proceduralist as needle advanced to keep needle tip in the US display
         2. Benefits – easier to obtain, allow for determination of lateral-medial needle position
         3. Risks – more difficult to determine needle depth

**Figure 13**: Short axis ultrasound guidance technique

- - 1.
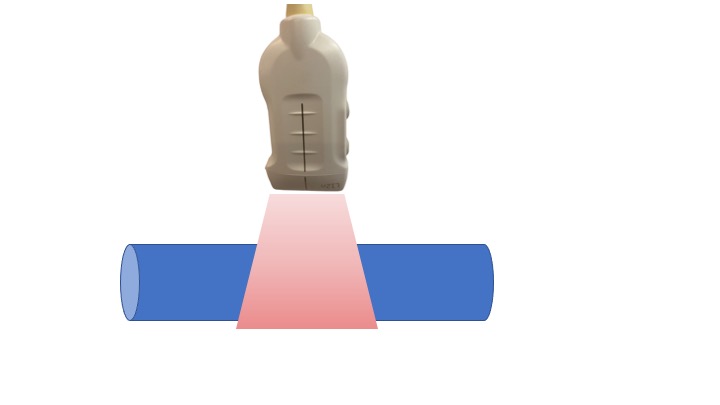
Long axis (in-plane, longitudinal) approach
       1. Probe remains still and needle advances
          1. If needle is not visible on US display, withdraw the needle, center on the probe and re-advance needle
       2. Benefits – needle depth clearly visualized
       3. Risks – more difficult to obtain

**Figure 14**: Long axis ultrasound guidance technique

- 1. Needle
     1. Any movement of the inserted needle can lacerate vessel and should NOT be done while the needle is inserted – withdraw the needle to the skin surface prior to any redirection of the needle
     2. **Femoral**
        1. Bevel = UP
        2. Angle = 20 to 30$^{\circ}$ to the skin
        3. Location = 1 to 2 cm inferior to the inguinal ligament, medial to the pulsation of the femoral artery
        4. Direction = umbilicus
     3. **IJ**
        1. Bevel = UP
        2. Angle = 30 to 45$^{\circ}$ to the skin
        3. Location = apex of the triangle formed by the heads of the SCM, lateral to the pulsation of the internal carotid artery
        4. Direction = ipsilateral nipple
     4. **Subclavian**
        1. Bevel = UP
           1. Rotating the bevel down once vessel is cannulated may help the wire to move into the innominate vein
        2. Angle = 10 to 15$^{\circ}$ to the skin
        3. Location = 1 to 2 cm inferior to the midpoint of the clavicle
        4. Direction = suprasternal notch
     5. Confirmation of venous position
        1. Qualitative – slow, non-pulsatile, dark red blood. Can be falsely positive in a patient who is hypotensive, hypoxemic or in cardiac arrest
        2. Blood gas analysis – specifically PaO2
        3. Pressure transduction – hook up pressure tubing to needle
  2. Guidewire
     1. **ALWAYS** maintain control of the guidewire during procedure to avoid intravascular foreign body
     2. The guidewire should pass smoothly and easily. Do NOT forcefully advance the guidewire
     3. Utilize US to confirm that the guidewire is in the vein PRIOR to passing the dilator to avoid inadvertent arterial injury
     4. The guidewire should retract from the catheter lumen easily. If resistance is noted do not tug or pull forcefully on the wire as it may severe distally and become an intravascular foreign body

**Figure 15**: Ultrasound image of guidewire in a simulated vessel in short (A) and long (B) axis. The guidewire is visible as a hyper-echoic structure (white arrow) within the anechoic vessel.


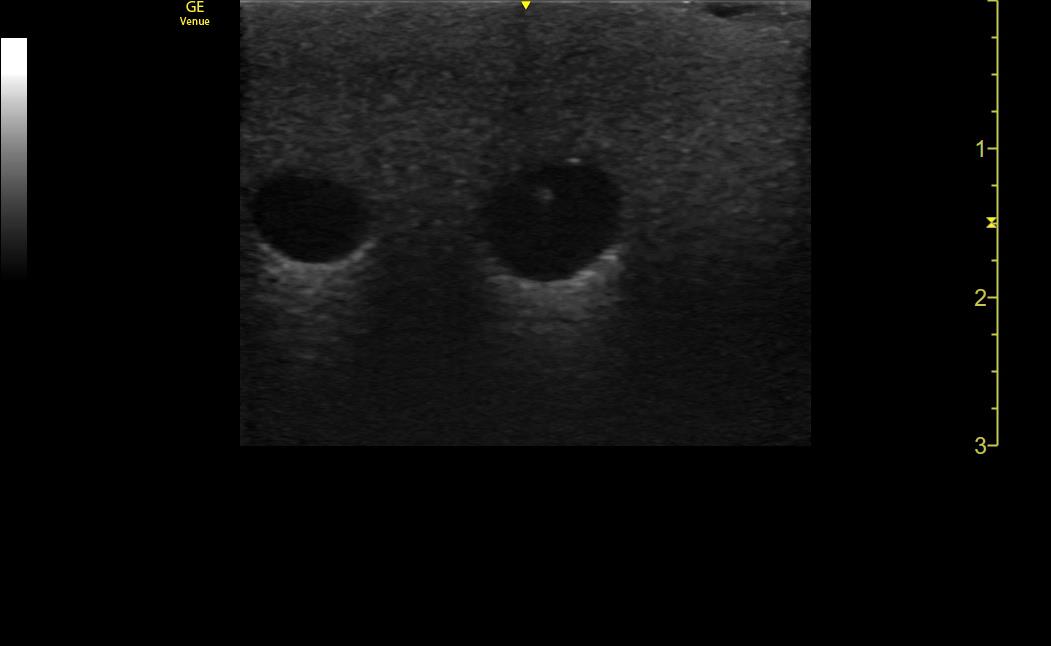

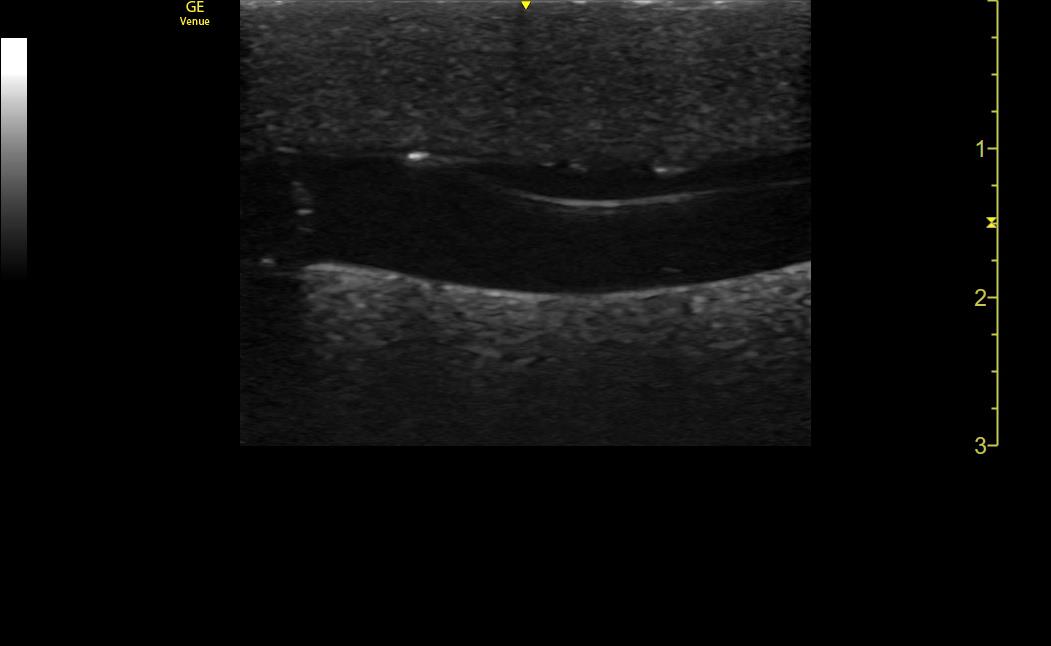


A

B

- 1. Incision
     1. Use #11 blade to make a stab incision in the skin at the entry site
  2. Dilator
     1. **DO NOT** hub the dilator! Only pass the dilator the depth necessary to reach the vein (can measure with US to be precise). Passing the dilator too far can lead to vascular injury.
  3. Catheter
     1. Test each lumen to ensure that each is able to flush and draw. If not, try adjusting the line position.

1. **Post-procedure**
   1. Confirmation
      1. Radiograph (chest or abdomen) = gold standard; should be performed prior to the used of all CVCs unless in an emergent situation
         1. **Femoral –** goal = tip in the IVC above the iliac vein confluence but below renal veins (L2)
         2. **IJ –** goal = tip just above the SVC-RA junction
         3. **Subclavian –** goal = tip just above the SVC-RA junction
      2. Alternatives
         1. US confirmation
            1. Direct visualization of the catheter in the vein
            2. Color Doppler flow or contrast flow with saline injection

**Figure 16**: Ultrasound image of catheter in a simulated vessel in short (A) and long (B) axis. The catheter is visible as a hyper-echoic structure (white arrow) within the anechoic vessel.


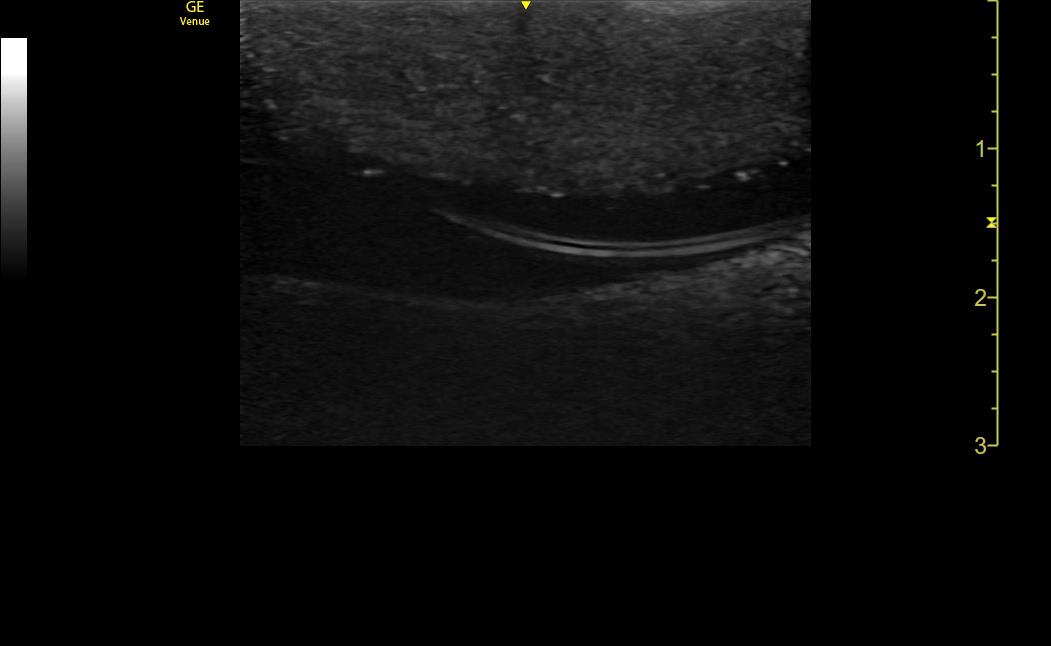

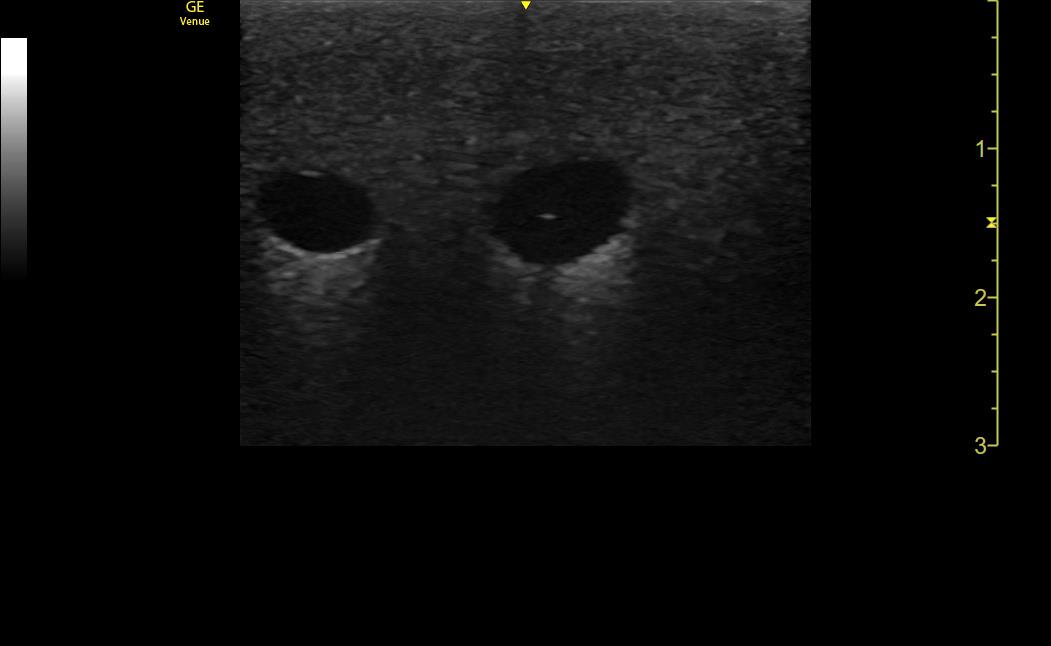


A

B

- - - 1. Pressure transduction
      2. Blood gas analysis
  1. Securing the line
     1. Use sutures to anchor the CVC hub to the skin. Sutures should be placed proximal to the hub.
     2. Number of knots should be 1 plus the size of the suture (e.g. 4 knots if using 3.0 silk)
     3. Cover the insertion point and hub with a transparent dressing
  2. Documentation – use the “PICU procedures” note template to document the procedure

1. **Complications**
   1. Bleeding
      1. Minimize blood loss by maintaining pressure on the site between steps of the procedure.
      2. If patient has evidence of coagulopathy, consider:
         1. Replacing with blood products to improve hemostasis
         2. Selecting a site at which direct pressure can be applied (i.e. femoral, IJ)
         3. Consulting surgery for CVC placement
   2. Hematoma
      1. Can be grossly visible or seen with US
      2. Presence can distort anatomy and/or make it difficult to pass guidewire or catheter
      3. Minimize risk by utilizing US guidance and maintaining pressure on the site between steps of the procedure and after any unsuccessful attempts
   3. Arterial puncture
      1. Bright red, pulsatile blood flow noted
      2. Hold pressure for 5-10 minutes if arterial puncture performed with needle
      3. If artery is dilated, hold pressure and consult vascular surgery for repair
   4. Bladder puncture
      1. Clear or yellow fluid noted from needle
   5. Arrhythmia
      1. May be noted during guidewire advancement in upper lines
      2. If note, withdraw guidewire (or catheter) until arrhythmia resolves
   6. Hemothorax/pneumothorax
      1. Potential complication of upper line placement but higher risk with subclavian
      2. Usually diagnosed on post-placement CXR
      3. Can use US to assess for either pneumothorax or hemothorax if high level of suspicion and waiting on CXR
   7. Malposition
      1. **Femoral**
         1. Past L2-L3 junction (i.e. too deep) 🡪 risk of renal vein thrombosis
         2. Cannulation of ascending lumbar vein can lead to spinal cord injury or ischemia. More common on the left but can occur on either side. Consider if any of the following are present and obtain lateral x-ray to confirm position:
            1. Inability to aspirate blood
            2. Lateral deviation of catheter
            3. Catheter path directly over the vertebral column
      2. **IJ** and **Subclavian**
         1. Past SVC-RA junction (i.e. too deep) 🡪 risk of arrhythmia, tamponade, valve injury or cardiac perforation
         2. Not central 🡪 increased risk of thrombosis
         3. If the catheter is directed cranially in the IJ, this should be removed and not used for central venous access.
2. **References**

1. Karapinar BB, Cura A. Complications of central venous catheterization in critically ill children. *Pediatr Int*. 2007;49(5):593-599. doi:10.1111/j.1442-200X.2007.02407.x

2. Scott-Warren VL, Morley RB. Paediatric vascular access. *BJA Educ*. 2015;15(4):199-206. doi:10.1093/bjaceaccp/mku050

3. Nifong TP, McDevitt TJ. The effect of catheter to vein ratio on blood flow rates in a simulated model of peripherally inserted central venous catheters. *Chest*. 2011;140(1):48-53. doi:10.1378/chest.10-2637

4. Citla Sridhar D, Abou-Ismail MY, Ahuja SP. Central venous catheter-related thrombosis in children and adults. *Thromb Res*. 2020;187(November 2019):103-112. doi:10.1016/j.thromres.2020.01.017

5. Shinohara Y, Arai T, Yamasita M. The optimal insertion length of central venous catheter via the femoral route for open-heart surgery in infants and children. *Paediatr Anaesth*. 2005;15(2):122-124. doi:10.1111/j.1460-9592.2005.01380.x

6. Maddali MM, Al-Shamsi F, Arora NR, Panchatcharam SM. The Optimal Length of Insertion for Central Venous Catheters Via the Right Internal Jugular Vein in Pediatric Cardiac Surgical Patients. *J Cardiothorac Vasc Anesth*. March 2020. https://linkinghub.elsevier.com/retrieve/pii/S1053077020302226.

7. Kim MC, Kim KS, Choi YK, et al. An estimation of right-and left-sided central venous catheter insertion depth using measurement of surface landmarks along the course of central veins. *Anesth Analg*. 2011;112(6):1371-1374. doi:10.1213/ANE.0b013e31820902bf

8. Shin HJ, Kim BG, Na HS, Oh AY, Park HP, Jeon YT. Estimation of catheter insertion depth during ultrasound-guided subclavian venous catheterization. *J Anesth*. 2015;29(5):724-727. doi:10.1007/s00540-015-2012-1

***All images and figures within this document were created by the authors***
